# Post-ICU Syndrome and the "3 Ds" in Geriatric Psychiatry: A Complicated Case Report of Major Depression With Psychosis in a 96-Year-Old Woman

**DOI:** 10.7759/cureus.76613

**Published:** 2024-12-30

**Authors:** Wejdan A Alshehri, Arij A Alzaidi, Nisreen Asraf

**Affiliations:** 1 Department of Family Medicine, Ministry of the National Guard-Health Affairs, King Abdulaziz Medical City, Jeddah, SAU; 2 Department of Family Medicine, Ministry of the National Guard-Health Affairs, King Abdulaziz Medical City, King Abdullah International Medical Research Center, Jeddah, SAU; 3 College of Medicine, King Saud Bin Abdulaziz University for Health Sciences, Jeddah, SAU

**Keywords:** delirium, dementia, depression, geriatric psychiatry, post-icu syndrome

## Abstract

In the growing field of geriatric psychiatry, the "3 Ds"-depression, dementia, and delirium-are a complex clinical challenge, especially in patients with medical comorbidities. This is a case report of a 96-year-old Saudi woman with chronic kidney disease, heart failure, and recurrent hyponatremia presented with worsening sleep, depression, persecutory delusions, and hallucinations following an intensive care unit (ICU) stay for urinary tract infection. Examination revealed cognitive decline and depressive symptoms, with sodium at 123 mmol/L. After a psychiatric evaluation, she was diagnosed with major depressive disorder with psychosis, likely exacerbated by hyponatremia and ICU-related stress. She was treated with sertraline, olanzapine, and sodium correction. One-month follow-up showed significant improvement in mood, sleep, and resolution of psychotic symptoms, restoring her quality of life. In conclusion, this case highlights the challenges between these conditions and the importance of comprehensive assessment in geriatric care.

## Introduction

In the growing field of geriatric psychiatry, the presence of depression, dementia, and delirium, generally referred to as the "3 Ds," poses a complex clinical challenge. This triad in the geriatric population usually coexists with other medical comorbidities, increasing the complexity of the patient's condition, which requires a special understanding and comprehensive assessment. Depression and delirium presentation in the geriatric population can be vague and sometimes misdiagnosed with dementia. Therefore, geriatric psychiatric conditions remain one of the top differential diagnoses in aging populations. Overall, the 3 Ds are common, disabling syndromes associated with poor prognosis in geriatrics [1].

Late-life depression is defined as depression occurring in patients older than 60 years of age, with a prevalence of 2% to 4% among geriatrics and 6% to 9% of those seen in primary health care clinics [2].

Delirium prevalence varies widely, with rates as low as 2% in outpatient settings but increasing with age, multimorbidity, and dementia [3]. Moreover, dementia prevalence in geriatrics above the age of 60 is around 5% to 7% and is recently considered the leading cause of dependence in the geriatric age group [4].

Post-intensive care syndrome (PICS) encompasses physical, neurocognitive, and psychological symptoms, many of which overlap with the "3 Ds," complicating diagnosis and management in geriatric patients. Post-intensive care syndrome includes a range of physical, neurocognitive, and psychological symptoms that commonly affect survivors of critical illness. These symptoms collectively impair physical function, mental health, and overall quality of life, persisting for months or even years after hospital discharge. Cognitive impairments are particularly prevalent and enduring among adult intensive care unit (ICU) survivors, affecting up to 60% across all age groups, and can be severe. Sleep disruption is linked to worse cognitive outcomes and increased functional decline in older ICU survivors. Additionally, up to 30% of ICU survivors experience long-term psychological symptoms stemming from acute stress, trauma, and anxiety during their ICU stay, including post-traumatic stress symptoms, anxiety, and depression. Several studies indicate that baseline physical function status is either unchanged or significantly declines post-discharge [5].

The literature review reported a lack of cases of the 3 Ds in geriatric psychiatry. Therefore, this case report aims to contribute to the growing literature on the 3 Ds in geriatric psychiatry by presenting a detailed clinical course, assessment, and treatment outcomes.

## Case presentation

A 96-year-old non-smoker Saudi woman widow illiterate patient resides in Jeddah city. She has nine children (five daughters and four sons) and 30 grandchildren. She lives with her son, who provides care and support for her.

She is known to have chronic kidney disease, atrial fibrillation, and heart failure with preserved ejection fraction (HFpEF) on a pacemaker, hypertension, bronchial asthma, and osteoporosis. She presented to our primary healthcare clinic with a history of inability to sleep well for the last two weeks, and she began to have persecutory delusions and visual and auditory hallucinations.

A full comprehensive history revealed that the patient's sleep deteriorated further, sleeping for only a few minutes, and started to express excessive sadness, lack of interest, crying spells, sleeping only a few hours per night, poor appetite, decreased concentration, restlessness, and feelings of anxiety and began to have persecutory delusions, visual and auditory hallucinations that her family wants to kill her and that they joke about her. She believed that they didn't love her. A few times, the family noted her asking, "Who's the person who entered into my language bag?".

She reported hearing devils and expressed fear, often looking around as if someone was present. She called her daughters and asked them to move her to another house, and she did the same with all of her sons and daughters. She told them that she would go out of the house as she felt scared of them and devils for the last three months without any clear trigger. Additionally, her family noted that she became slower in speech and experienced a decline in cognitive function. There were no death wishes or other safety concerns.

During her ICU stay, she was diagnosed with a multidrug-resistant (MDR) urinary tract infection and hypovolemic hyponatremia, which may have contributed to her subsequent neuropsychiatric symptoms. The patient received multiple intravenous antibiotics, norepinephrine, and intravenous fluids during that ICU stay. She was not connected to mechanical ventilation at any point during her ICU stay. The patient remained in the ICU for a total of four days before being discharged. Since then, she has become fully dependent on her family, using diapers, and needs assistance in mobilization.

After discharge, the patient had a history of recurrent emergency department visits, complaining of not sleeping well and having persecutory delusions and visual and auditory hallucinations every time diagnosed as hyponatremia. Appropriate treatment was given, and she was discharged home without any follow-up appointments.

Upon assessment in our clinic, she was vitally stable, with a heart rate of 63 beats/min, blood pressure of 108/62 mmHg, respiratory rate of 20 breaths/min, oxygen saturation of 97% on room air with no signs of respiratory distress, and a temperature of 36.5°C. She was walking in a wheelchair. She was conscious oriented to place but disoriented to person and time. She had a score of four points, revealing a negative dementia screening on the Mini-Cog test. The mini-mental state examination (MMSE) and Geriatric Depression Scale (GDS) scored 25/30 and 11/15, respectively. Lung examination showed equal bilateral air entry with no added sounds. Cardiac auscultation showed normal first heart sound (S1) and second heart sound (S2) with a systolic murmur and no lower limb edema.

**Figure 1:**
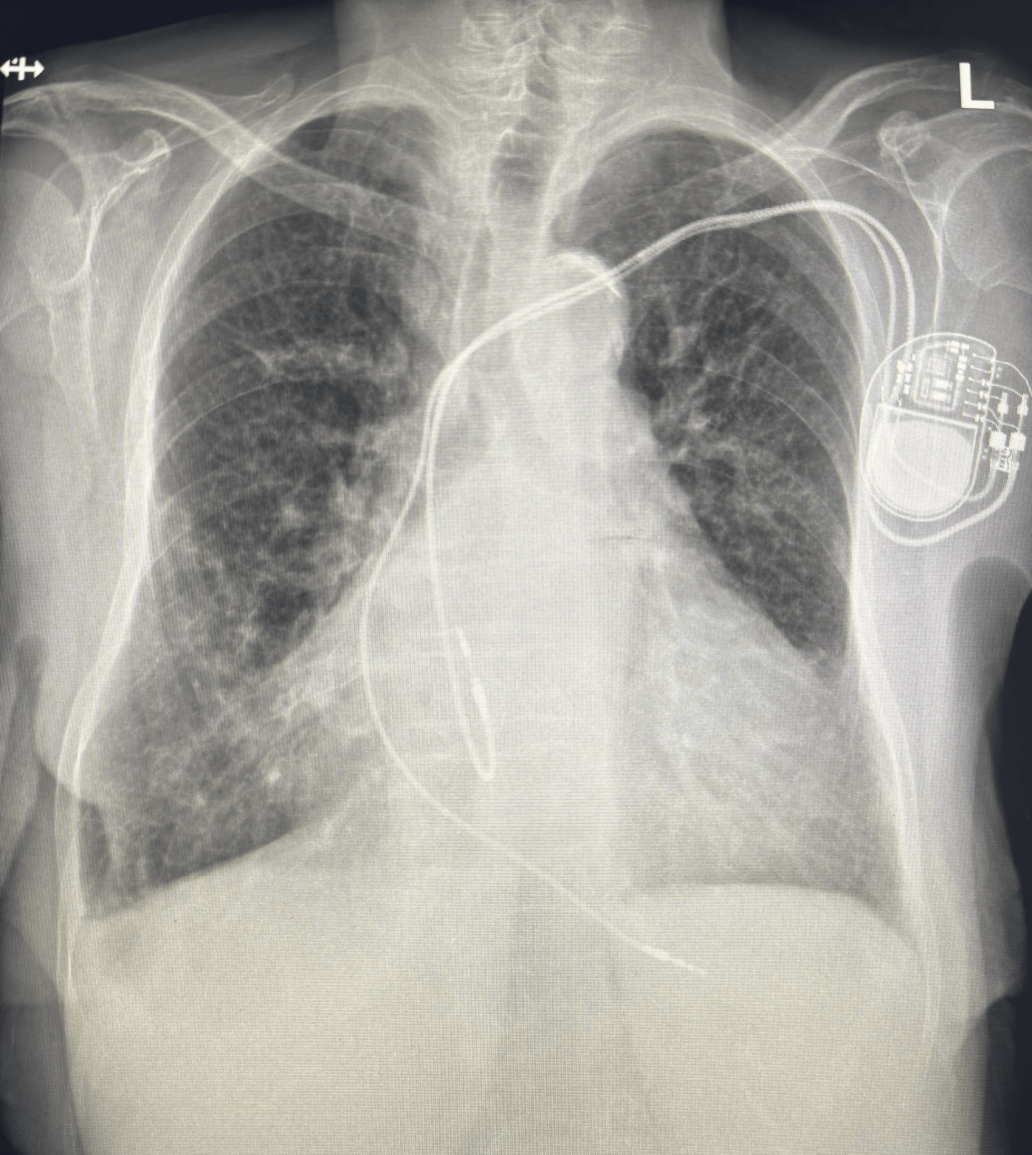
Chest x-ray shows the PC maker in place; otherwise, it is unremarkable for any other pathological findings. PC: pacemaker.

**Figure 2:**
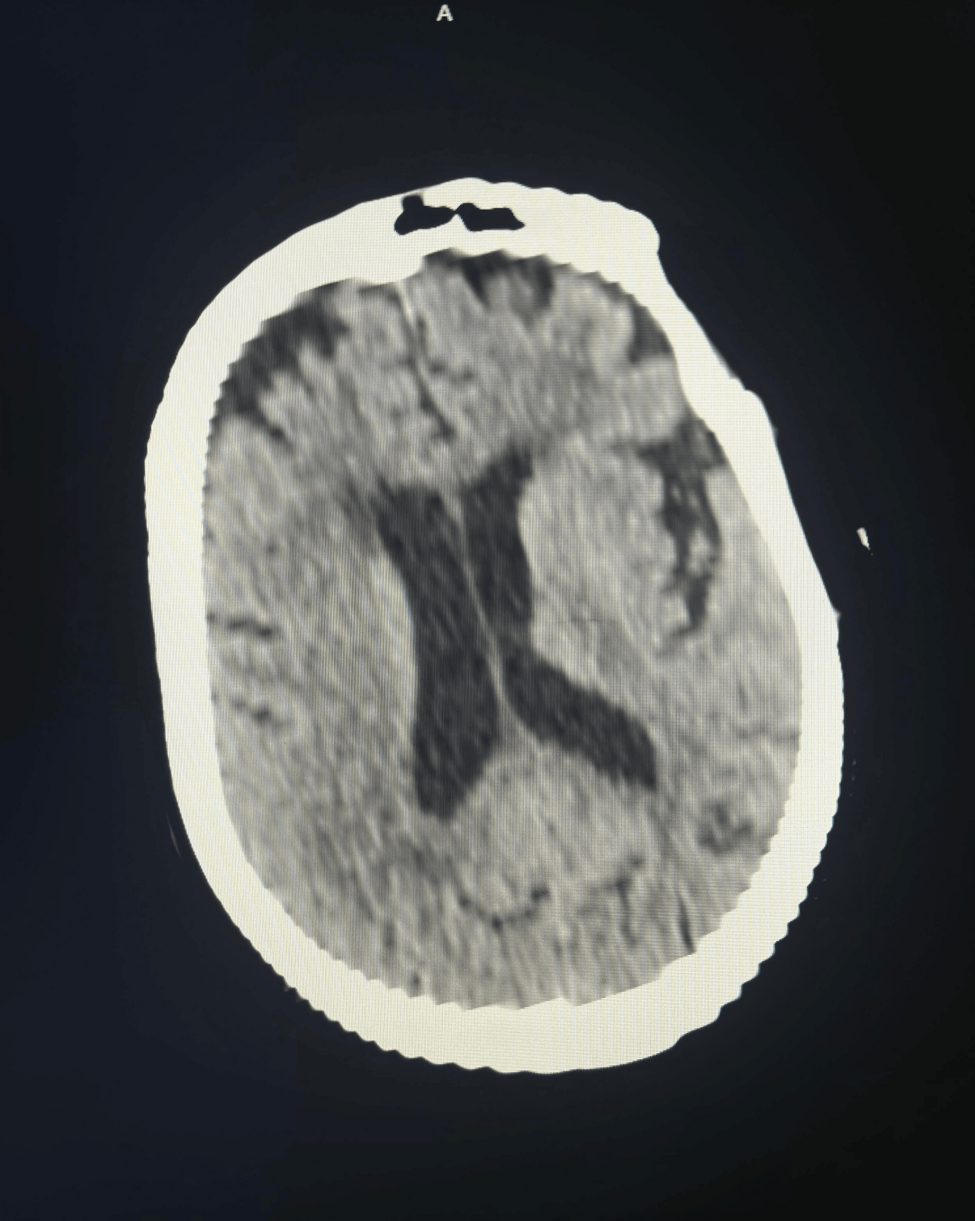
Head computed tomography cross-sectional image shows age-related degenerative changes and mild brain atrophy, otherwise unremarkable for any other pathological findings.

A comprehensive set of laboratory tests, including complete blood count with differential, renal profile, liver profile, and urinalysis, was ordered. Upon evaluation, most results were within her baseline. However, the sodium level was noted to be 123 mmol/L, below the standard reference range of 135-145 mmol/L (Table 1). Radiological investigations and an electrocardiogram (ECG) were also conducted, revealing unremarkable findings. Also, chest x-ray and brain computed tomography showed no abnormality (Figures 1, 2).

**Table 1:** Laboratory characteristics. WBC: white blood count, CO_2_: carbon dioxide, CK: creatinine kinase, ALT: alanine aminotransferase, AST: aspartate aminotransferase, ALP: alkaline phosphatase, GGT: gamma-glutamyl transferase, CRP: C-reactive protein, PT: prothrombin time, PTT: partial thromboplastin time, INR: international normalized ratio, LDH: lactic dehydrogenase, GFR: glomerular filtration rate.

Laboratory variable	Reading	Reference range	Laboratory variable	Reading	Reference range
WBC	7 K/uL	4.5-11.5 K/uL	GGT	25 U/L	5-40 U/L
Hemoglobin	10.3 g/dL	14-18 g/dL	CRP	30 mg/L	<3 mg/L
Platelets	195 K/uL	150-450 K/uL	Albumin	37 g/L	40.2-47.6 g/L
Lactate	1.9 mmol/L	0.4-2 mmol/L	PT	15 seconds	9.4-12.5 seconds
Troponin I	21.6 ng/L	<20 ng/L	PTT	47 seconds	25-36.5 seconds
Glucose	6.5 mmol/L	4.4-7.8 mmol/L	INR	1.3 ratio	0.85-1.3 ratio
PH	7.42	7.35-7.45	LDH	244 U/L	100-240 U/L
pCO_2_	39 mmHg	35-45 mmHg	Sodium	123 mmol/L	135-145 mmol/L
Bicarbonate	29 mmol/L	20-28 mmol/L	Potassium	3 mmol/L	3.5-5.1 mmol/L
Base deficit	1.9 mmol/L	>-4 mmol/L	Chloride	81 mmol/L	98-107 mmol/L
Folate	36.5 nmol/L	4.5-45.3 nmol/L	Magnesium	0.68 mmol/L	0.7-1 mmol/L
Vitamin B12	693 pmol/L	118-701 pmol/L	Calcium	2.3 mmol/L	2.2-2.6 mmol/L
CK	31 U/L	22-198 U/L	Phosphorus	1 mg/dL	2.5-4.5 mg/dL
ALT	7 U/L	7-55 U/L	Urea	12.1 mmol/L	2.5-6.4 mmol/L
AST	25 U/L	8-33 U/L	Creatinine	86 umol/L	53-115 umol/L
ALP	70 IU/L	44-147 IU/L	GFR	53 ml/min	>90 ml/min

Major depressive disorder with psychosis with dementia and delirium was suspected as the most likely presumptive diagnosis, and a psychiatry consultation was obtained. Therefore, the patient was started on sertraline 25mg once daily (OD) and olanzapine 2.5 mg at bedtime (HS). Simultaneously, the patient was admitted under the care of internal medicine as a case of asymptomatic hyponatremia. Following a two-day hospital stay, during which appropriate treatment was given, the patient exhibited satisfactory improvement and was discharged home in a well-conditioned state.

After one month of follow-up, the patient showed much improvement in her depressive symptoms, was able to relax and enjoy her time, had no more crying spells, improved appetite, and seems happier now and reactive. No more irritability or restlessness. The patient was sleeping well after the medication was initiated. There were no more psychotic symptoms and no persecutory ideas.

## Discussion

This case report presents a case with a chief complaint of sleep disturbances, an indicator of an underlying psychiatric illness that revealed itself upon the unfolding of the case. Previous studies have highlighted the association between cardiovascular diseases and an increased risk of depression in older adults [6,7]. Furthermore, the complex interplay between chronic medical conditions, such as heart failure, and the development of cognitive impairment and psychiatric symptoms necessitates a holistic approach to clinical management [8,9].

Delirium is the most prominent independent risk factor for cognitive impairment, with up to 40% of patients during hospitalization deemed preventable. The best evidence for the management and prevention of delirium is based on multicomponent bundles of care, as there is no currently FDA-approved treatment for delirium [10]. The most effective strategy to manage delirium includes non-pharmacological multicomponent intervention bundles such as the ABCDEF bundle. Each letter addresses a component of care associated with attempts to maintain brain health in the ICU: (A) assess, prevent, and treat pain; (B) perform both spontaneous awakening and spontaneous breathing trials; (C) choose analgesic and sedative medications thoughtfully; (D) perform frequent Delirium assessments and provide appropriate prevention and management; (E) engage in early mobility and exercise; and (F) encourage family engagement. The management of consequences following critical diseases is based on selecting sedative and analgesic agents, thoughtfully performing frequent delirium assessments, and providing suitable treatment and prevention for the patient. Several studies that adopted the bundle for prevention and management revealed a reduction in delirium, mortality, and comas, with high compliance with all bundle components [11,12]. However, the full implementation of such bundles is not possible among older adults. 

Major depression is common in elderly patients, especially those who were recently admitted to the hospital. When selecting selective serotonin reuptake inhibitors (SSRIs) for elderly patients with depression, recommendations from the American Geriatrics Society (AGS) highlight several important factors. The AGS guidelines emphasize the importance of selecting SSRIs with favorable pharmacokinetic profiles, minimal drug interactions, and a low potential for adverse effects in older adults. Sertraline and citalopram are frequently recommended due to their relatively favorable safety profiles and effectiveness in this demographic. Sertraline is particularly favored for its lower risk of drug interactions and side effects such as sedation compared to other SSRIs. Although citalopram is effective, its dosage must be carefully managed because of the risk of QT prolongation at higher doses. These considerations underscore the importance of individualizing treatment based on the patient's overall health status, concurrent medications, and potential for drug interactions [13].

The comprehensive geriatric assessment (CGA) is designed to evaluate the overall health status of elderly patients and guide tailored interventions. Key components of the CGA include medical, cognitive, mental health, nutritional, social, functional, mobility, and environmental assessments, along with a thorough review of medications and advanced care planning [14]. Sleep may play a crucial role in recovery, as sleep disruption is associated with worse cognition in older ICU survivors and worsened functional decline [15-17]. This may explain the improvement of sleep of our patient after the management of her condition.

Geriatric patients are at an increased risk of major depression due to the presence of comorbid conditions. Comprehensive assessment is crucial for complex geriatric cases for precise diagnosis and management. The depression may be worsened following hospital admission and the requirement of medical care, especially following admission to ICU at post-ICU syndrome results in psychological impact in addition to the impact of critical illness. Therefore, the pharmacological therapy of depression can be effective in reducing depression symptoms as well as associated hallucinations and cognitive changes. However, prescribing medication for this population should be performed based on AGS guidelines; therefore, the treatment must be individualized based on the patient's overall health status and other considerations. The correct diagnosis among the geriatric population is a crucial step for proper management. This correct diagnosis mainly depends on an interdisciplinary collaboration between specialties to improve the outcomes.

## Conclusions

The diagnostic challenges and therapeutic interventions discussed here shed light on the cornerstone role of family medicine physicians in identifying changes in mental status in outpatient settings through using a comprehensive and interdisciplinary approach. Some learning points worth sharing with readers of this case. First, depression diagnosis among the geriatric population is challenging and requires comprehensive assessment. Second, psychological assessment following hospital admission, especially ICU admission, is required to look for post-ICU syndrome. Finally, cooperation between specialists is necessary for the adequate and proper management of geriatric cases for a favorable outcome.

## References

[1] Weng CF, Lin KP, Lu FP (2019). Effects of depression, dementia and delirium on activities of daily living in elderly patients after discharge. BMC Geriatr.

[2] Dharia S, Verilla K, Breden EL (2011). The 3 D's of geriatric psychiatry: depression, delirium, and dementia. Consult Pharm.

[3] Setters B, Solberg LM (2017). Delirium. Prim Care.

[4] Alkhunizan M, Alkhenizan A, Basudan L (2018). Prevalence of mild cognitive impairment and dementia in Saudi Arabia: a community-based study. Dement Geriatr Cogn Disord Extra.

[5] Marra A, Pandharipande PP, Girard TD (2018). Co-occurrence of post-intensive care syndrome problems among 406 survivors of critical illness. Crit Care Med.

[6] Alexopoulos GS (2005). Depression in the elderly. The Lancet.

[7] Byers AL, Yaffe K (2011). Depression and risk of developing dementia. Nat Rev Neurol.

[8] Cohen-Mansfield J, Mintzer JE (2005). Time for change: the role of nonpharmacological interventions in treating behavior problems in nursing home residents with dementia. Alzheimer Dis Assoc Disord.

[9] Vann Jones SA, O'Brien JT (2014). The prevalence and incidence of dementia with Lewy bodies: a systematic review of population and clinical studies. Psychol Med.

[10] Devlin JW, Skrobik Y, Gélinas C (2018). Clinical practice guidelines for the prevention and management of pain, agitation/sedation, delirium, immobility, and sleep disruption in adult patients in the ICU. Crit Care Med.

[11] Barnes-Daly MA, Phillips G, Ely EW (2017). Improving hospital survival and reducing brain dysfunction at seven California community hospitals: implementing PAD guidelines via the ABCDEF bundle in 6,064 patients. Crit Care Med.

[12] Pun BT, Balas MC, Barnes-Daly MA (2019). Caring for critically ill patients with the ABCDEF bundle: results of the ICU liberation collaborative in over 15,000 adults. Crit Care Med.

[13] 2019 American Geriatrics Society Beers Criteria® Update Expert Panel (2019). American Geriatrics Society 2019 updated AGS Beers Criteria® for potentially inappropriate medication use in older adults. J Am Geriatr Soc.

[14] 2023 American Geriatrics Society Beers Criteria® Update Expert Panel (2023). American Geriatrics Society 2023 updated AGS Beers Criteria® for potentially inappropriate medication use in older adults. J Am Geriatr Soc.

[15] Wolters AE, van Dijk D, Pasma W (2014). Long-term outcome of delirium during intensive care unit stay in survivors of critical illness: a prospective cohort study. Crit Care.

[16] Gunther ML, Morandi A, Krauskopf E (2012). The association between brain volumes, delirium duration, and cognitive outcomes in intensive care unit survivors: the VISIONS cohort magnetic resonance imaging study. Crit Care Med.

[17] Hope AA, Morrison RS, Du Q, Wallenstein S, Nelson JE (2013). Risk factors for long-term brain dysfunction after chronic critical illness. Ann Am Thorac Soc.

